# Intelligence

**Published:** 1832-05

**Authors:** 


### INTELLIGENCE.

---

### KING'S COLLEGE, LONDON.

*Report presented by the Council to the Annual General Court of the Governors and Proprietors, on Wednesday, the 11th of April, 1832.*

THE Council, in commencing their report of the proceedings of the last year, desire to congratulate the General Court on the opening of the College, for the purposes of education, with a degree of success fully commensurate with the expectations which have been formed by the friends and supporters of the institution.

The public opening of the College took place on the 8th of October last, when his Grace the Visiter, several Members of the Council, the Principal and Professors, together with a large number of Proprietors and friends of the Institution, attended Divine Service in the College Chapel. The Lord Bishop of London preached on the occasion, and the Principal delivered an address.

On Monday the 10th of October, the regular business of the College commenced; and it has since been carried on without intermission, except during the vacation of about one month at Christmas.

| | |
|---|---|
| The whole number of Students in all departments entered on the books of the College, up to the present time, is ................ 764 | |
| Of these, the Students for General Education, in the Senior Department | 66 |
| Occasional Students in different departments of general Literature and Science ........................................ | 149 |
| Regular Students for the General Course in the Medical Department .. | 48 |
| Occasional Students in the various departments of the Medical School.. | 107 |
| Pupils in the Junior Department for General Education ............ | 162 |
| | 532 |

The remainder of the students entered, belong to the Medical Department, and consist chiefly of persons who had previously attended the Professors at other Lecture Rooms.

The gratifying fact, that within the short period of six months from the opening of the College, more than 750 Students have become partakers of the advantages which it affords, is an encouraging evidence of the approbation with which the public regard, as well the principle on which the College was founded, as the plan on which it is conducted; and, at the same time, holds out the promise of future usefulness on a more extended scale.

Many liberal donations of books, &c. to the Library, and of Botanical and other specimens, Anatomical preparations, and various articles for the Museum, have been made by Proprietors and other individuals; and it is especially incumbent on the Council to record the mark which His Majesty has been graciously pleased to give of his royal favor towards the Institution, of which he is the Patron, by presenting to it a very ingeniously constructed model of the human frame, intended to assist the studies of medical pupils. The Council take this opportunity of mentioning, that, as the rooms for the reception of such donations are now ready, all contributions of the foregoing description will be highly acceptable, and will be gratefully recorded.

In adverting to the present state of the Building, the Council beg to call to the recollection of the General Court the plan of proceeding which was approved by them in the last year; namely, that in the first instance, those portions of the structure should be completed which would be immediately required for the purposes of education, and that the remainder should be postponed till after the opening of the College. In pursuance of this plan, the interior fittings of several of the apartments in the portion of the building already raised, have not been completed; and the other portion, which will complete the river front, has not yet been commenced.

To this point it is necessary to direct the special attention of the Court.

The ground on which the College is erected, having been granted by his Majesty's government on the express condition that the river front should be completed at a period not later than the month of June, 1834, the Council are desirous of proceeding immediately with this part of the work. But, on considering the present state of their funds, they regret to find that, in consequence of many sums being withheld by a number of the original subscribers, amounting on the whole to more than thirteen thousand pounds, the means remaining at their disposal are wholly inadequate to the execution of the work; and that therefore they must appeal to the liberality of the friends of the institution for the supply of the necessary resources.

Sir Robert Smirke, the architect, has estimated the expense of completing the Terrace, and River front, at about the sum of £12,000, independently of the principal part of the interior fittings.*

The Council, therefore, think it right to suggest, that books should immediately be opened for raising a fund in the way of donations, and of subscriptions for shares of £100 each, towards erecting that part of the building which will form the River front, and fitting up those Lecture Rooms, and other apartments, which, it is expected, will soon be required for the purposes of education; the donations and subscriptions to convey, of course, the same privileges of Proprietorship with those already received.

In submitting to the General Court the Financial Report for the past year, the Council have great satisfaction in noticing the munificent legacy of £1000, left to the College, by the late Mrs. Duppa, whose attachment to the principles upon which this institution was founded, was evinced by many acts of liberality during her lifetime. The Council have also the pleasure of recording many additional donations, and subscriptions for shares.

The Council cannot refrain from renewing the expression of their regret at the non-payment of so many contributions originally promised, which amount, on the whole, to the large sum already mentioned; and they are unwilling to abandon the hope, that the Subscribers will seriously consider whether they are justified in withholding sums to which they affixed their names, and on the faith of which the engagements for the buildings were made.

At all events, the zeal and liberality evinced by the numerous friends of this institution, encourage the Council to entertain a confident hope that sufficient means will be placed at their disposal to enable them, by fulfilling the condition on which the site was granted, to place the possession of the building on a footing of permanent security, at the same time that they complete one of the finest ornaments of the metropolis.

Signed by order of the Council,

H. Smith, Secretary.

Of such an institution as this, we feel that something better than a mere formal announcement of its last public proceedings is required at our hands; for its scheme does not stop short with the endeavour to impart a good general education to its *alumni*, but extends to an object of more than every-day utility, that of attempting to make sound principles travel hand in hand with the advance in scientific attainments. It is obvious that such a scheme and such an institution must come recommended to the notice of the individual practitioner, no less than to that of society at large, by a claim, of which no parent or reasonable being amongst us can refuse to admit the deep and extensive importance, and in which will be found the motive which has impelled us to avail ourselves, somewhat at length, of the information which we are enabled to collect from former Reports, as well as that just put into circulation, and which we subjoin.

The plan of this College was projected in June 1828: the limit of £100,000 of subscriptions, at which the provisional committee were empowered to commence active operations, was obtained in the course of the following December; the present site was presented by his Majesty's government in June 1829; the late King sanctioned the plan by a royal charter, in the subsequent month of August. The public opening took place on the 8th of October last, in the presence of the Archbishop of Canterbury, several distinguished prelates and noblemen, and a most respectable assemblage of the proprietors and friends of the institution, who were collected in the chapel attached to it. The regular busi-

* It appears from the Report of 1830, that for attaining all the objects contemplated in the foundation of the College, it was estimated that a further sum of above £50,000 may ultimately be required.

ness of the College began on the following Monday; and it has since been carried on without intermission, excepting during the last Christmas vacation.

On reviewing the detail of the number of students entered, in all departments, on the books of the College up to the present time, we are ready to admit that its directors are justly entitled to congratulate the proprietors on the high degree of success which has attended its early operations. This detail informs us that the number of students entered is 764, many of whom are matriculated and attend all the classes, although, of course, they are enumerated but once. This, as the last Report observes, "is an encouraging evidence of the approbation with which the public regard, as well the principle on which the College was founded, as the plan on which it is conducted; whilst, at the same time, it holds out the promise of future usefulness on a more extended scale." It is equally satisfactory to learn, from the same authority, that the progress which the pupils of the College are making, and the punctuality with which they attend the respective Courses, are dwelt upon in highly favorable terms in the reports made by the principal and professors.

In addition to the collections formed out of the funds of the Institution itself, we observe that many liberal and valuable donations of books, charts, &c. to the library, and of botanical and other specimens, anatomical preparations, and a variety of objects in natural history for the museum,* have been presented both by the proprietors and individuals; and his present Majesty has, we are happy to find, not merely bestowed his nominal patronage, but evinced the continued interest which he takes in the welfare of the College, by presenting to it the first of Dr. Auzoux's ingenious models of the human frame which has reached this country.

Amidst these topics for congratulation, we remark, with great regret, that the defalcation of funds, originating in the secession of several subscribers who had pledged themselves to support the College at its outset, amounts to so considerable a sum as £13,000; and, although it will not arrest its academical operations, that it is prejudicial enough to interfere with the keeping of the engagement which was contracted with government, for the completion of the river front of Somerset House, in consideration of the grant of the site. No just or reasonable grounds, so far as we have been able to learn, have been assigned for this extraordinary secession: but, be this as it may, those whose liberality has already been so conspicuous in bringing the institution into its present efficient state, have again come forward to begin the good work of removing this temporary difficulty, to put the finishing hand to one of the finest ornaments of the metropolis, and complete the other objects for which the institution was founded. To doubt the influence of the example which has thus been anew held out, would be to doubt the existence of that public spirit which has hitherto never failed to patronize every undertaking that has had the welfare of society at large, and that of the rising generation in particular, for its sole and momentous object.

---

### PRIZE QUESTIONS.

At a Special Meeting of the Council of the Medico-Botanical Society of London, holden on Friday, the 6th day of January, 1832, HUMPHREY GIBBS, Esq., F.H.S., Treasurer, in the chair,

It was resolved, That the gold medal of the Society should be offered for the

* Amongst these are a cabinet of Materia Medica, from Sir H. HALFORD; an extensive and choice collection of Botanical Specimens, collected in the East, by Dr. WALLICH; a collection of Fossils and Minerals, brought from Repulse Bay and Melville Island, by Lord BEXLEY; of Reptilia, &c. from Penang, by Mr. KERR; and of Snakes, &c. from the Cape, by Captain RONALD; besides numerous articles and books from Professors GREEN, BURNETT, MAYO, and others.

best Essay in the English, French, German, or Latin language, on the question, "What is the vegetable substance which could be employed with the greatest success in Cholera?" And that the silver medal of the Society should be offered for the best Essay "On the analysis of any vegetable substance, the proximate principle of which may be employed in the cure of disease," provided that such Essay possesses sufficient merit; and that they should be received till the close of the year 1833, and that the medals should be bestowed at the anniversary, January 16th, 1834.

And it was further resolved, That as the question, "What is the vegetable substance which could be employed with success in Hydrophobia?" is a subject of great importance, that the time for receiving Essays on the same be extended to the last day of December, 1832.

That each Essay shall be accompanied by a sealed paper, containing the names and address of the author, and marked in the same manner as the Essay; and that each Essay to which a medal is not awarded, shall, according to the wish of the author, be restored to him, or submitted to the Council, in order to its being read at a general meeting.

By order of the Council,

G. G. Sigmond, Secretary.

---

*Anatomical Model, invented by* M. Auzoux.

With whatever elegance and fidelity anatomical plates, or wax models, may be executed, they must be very inferior for the purposes for which they are intended to this curious, and even wonderful, contrivance of M. Auzoux. It consists of the representation of an athletic man, about five feet six inches in height, standing upon a wooden frame in a well-chosen position. The figure may be turned in any direction, as the feet revolve upon a pivot. The integuments are supposed to be removed, and the spectator is immediately struck with a beautiful display of the first layer of well-developed muscles, which are as clearly and precisely presented to view as if they had been prepared for inspection by the knife of the most dexterous anatomist. Each muscle may be separately removed with the utmost facility, and every part of the structure of the human frame successively exposed to inspection; all the muscles of the body, the blood-vessels, nerves, and viscera, are represented with the utmost fidelity in form, colour, and their respective connexions with each other. The representation of the heart, kidney, and the intricate anatomy of the head and throat, attracted our particular attention. The various parts which form the mouth, the pharynx, larynx, and nasal fossa, are accurately copied from nature; and the brain, spinal marrow, and great sympathetic nerve, are represented with equal fidelity. This figure is made of a kind of paste, which, when first made, may be moulded to any shape, and when dry, it becomes hard and very durable. Each part is numbered, so that, when the dissection has been completed, there is no difficulty in replacing the whole of the parts in their proper situation.

M. Auzoux himself does not presume that the study of his model can supersede the necessity for dissection; but there can be no doubt that, by its assistance, the student may acquire a perfect knowledge of anatomy, at much less expense of time than he could by anatomical lectures or dissection alone. One great advantage this contrivance offers, is that the relative situation of every part of the human body may be repeatedly and deliberately viewed, so that this most important part of the science of anatomy may be indelibly fixed upon the memory.

M. Auzoux has exhibited his model at the Royal Institution, the Westminster Medical Society, and the Harveian Society, and received the most flattering marks of approbation. We understand that his Majesty has presented the King's College with one of the models.

### LITERARY INTELLIGENCE.

In the press, A Practical Treatise on Diseases of the Pelvic Viscera: Part I. By HERBERT MAYO, F.R.S., Surgeon to the Middlesex Hospital, &c.

We have more than once expressed our gratification at the rapid improvements that have recently been made in the art of veterinary surgery, and we are glad to find that Mr. SCHLOSS, of St. Martin's lane, is about to publish a translation from the German of "the Anatomy of the Horse," by Professor GUILT, of Berlin, which cannot fail to be a valuable addition to the library of the veterinary surgeon and student. The work is to be dedicated to Professor COLEMAN. We have seen the plates, but we prefer giving their character from the report of several eminent veterinary surgeons, who assure us that they are as correct in detail as they are elegant in their execution. We shall give an account of this work when the translated text is published.

APRIL 5.

Benjamin Meredith Bradford, of Chepstow
Joseph Cunnington, King's Cliffe
Freeman Eaton, Grantham
Carsten Holthouse, Edmonton
John Stanton, Bristol
George Turner, Lofthouse
Edward Williams, Caerphilly

APRIL 12.

Matthew Brunskill, of Kirkby Stephen
Thomas Clarkson, West Welton, Yorkshire
Jallard Jennings Edenborough, London
Thomas Grundy, Warrington
Henry Hall, Ashmore, Dorsetshire
William Haine Maunder, Honiton
Richard Pullen, Leeds
Joseph Walker Ratcliffe, Wrexham
Henry Thomas, Halifax
John Whyte, Munkirk

APRIL 19.

Thomas Evans Brinsden, of Marlborough
Augustus Cooke, Yortworth
Richard Henry Crow, Wrotham
Benjamin Davies, London
David Hartley, Clifton
Thomas Edward Osmond, Thorp, Essex
George Ord, Newcastle
William Rayner, Lincoln
William Richardson, London
John Skevington, Ashbourn

APRIL 26.

John Bartlett, of Great Bedwin
William Robert Clavey, Bath
Richard Ebsworth, Shillingford
George Ellerton, Kippax
John Freeland Fergus, Newcastle on Tyne
Edward Hodges, Leicester
John Jobson, Newcastle on Tyne
George Mousley, Chilcote
Samuel Richards, London
Zachariah Robert Stewart, Southwark
Joseph Sadler, Barnsley.

### METEOROLOGICAL JOURNAL,

By Messrs. Harris and Co. Mathematical Instrument Makers, 50, High Holborn.

| | Moon. | Rain gauge. | Thermometer. | | | Barometer. | | De Luc's Hygrometer | | Winds. | | Atmospheric Variation. | | |
|---|---|---|---|---|---|---|---|---|---|---|---|---|---|---|
| Mar. | | | 9a.m. | max. | min. | 9a.m. | 10p.m. | 9a.m. | 10p.m | 9 a.m. | 10p.m. | 9 a. m | 2 p.m. | 10 p.m. |
| 20 | | | 47 | 49 | 44 | 29.40 | 29.72 | 54 | 54 | NNW | NW | fine | fine | fine |
| 21 | | | 50 | 54 | 42 | .81 | .93 | 56 | 60 | W | SW | | cloudy | |
| 22 | | | 46 | 48 | 38 | .97 | .92 | 60 | 58 | WSW | WSW | cloudy | fine | |
| 23 | | | 41 | 44 | 37 | .74 | .67 | 55 | 53 | WSW | NW | | | |
| 24 | ☾ | | 42 | 45 | 37 | .67 | .75 | 52 | 55 | NW | N | fine | sn. & sle. | |
| 25 | | .02 | 40 | 45 | 37 | .92 | .98 | 55 | 55 | NE | N | | fine | |
| 26 | | | 45 | 50 | 41 | 30.00 | .92 | 55 | 56 | NW | WNW | cloudy | | |
| 27 | | | 48 | 51 | 40 | 29.82 | .87 | 56 | 56 | NNW | ENE | fine | | |
| 28 | | | 45 | 48 | 33 | .92 | .88 | 56 | 53 | SE | SSE | | | |
| 29 | | | 45 | 52 | 39 | .80 | .80 | 52 | 52 | E va. | E | | | |
| 30 | | | 43 | 48 | 37 | .80 | .75 | 55 | 57 | NE | SE | | | |
| 31 | | | 46 | 48 | 42 | .63 | .56 | 57 | 56 | ESE | N | cloudy | cloudy | |
| April 1 | ● | | 47 | 52 | 45 | .73 | .87 | 55 | 53 | NW | NW | fine | fine | |
| 2 | | | 50 | 54 | 42 | .96 | 30.04 | 53 | 52 | W | SW | | | |
| 3 | | | 55 | 59 | 46 | 30.22 | .27 | 51 | 52 | WSW | WSW | | | |
| 4 | | | 54 | 60 | 53 | .35 | .34 | 52 | 52 | SSE | SE | | | |
| 5 | | | 59 | 66 | 45 | .27 | .22 | 56 | 56 | SE | ESE | foggy | | |
| 6 | | | 55 | 56 | 38 | .18 | .16 | 55 | 57 | NE | ENE | | | |
| 7 | | | 45 | 50 | 40 | .07 | 29.98 | 57 | 57 | NE | ESE | | | |
| 8 | ☽ | | 46 | 53 | 42 | 29.96 | 30.00 | 56 | 56 | ESE | ENE | fine | | |
| 9 | | | 47 | 54 | 38 | 30.04 | .03 | 56 | 54 | NE | ENE | | | |
| 10 | | | 49 | 56 | 38 | .04 | 29.98 | 51 | 51 | ENE | NE | cloudy | | |
| 11 | | | 47 | 51 | 39 | 29.99 | .88 | 48 | 52 | ENE | E | fine | | |
| 12 | | | 42 | 51 | 42 | .76 | .75 | 57 | 56 | E | NE | rain | | cloud |
| 13 | | .04 | 50 | 54 | 41 | .74 | .86 | 55 | 53 | SE | S | fine | | fine |
| 14 | | | 48 | 56 | 39 | .96 | .96 | 50 | 50 | SE | NNE | | cloudy | cloudy |
| 15 | ○ | | 46 | 54 | 45 | .93 | .85 | 52 | 52 | NNW | NNW | | fine | rain |
| 16 | | .08 | 49 | 54 | 46 | .82 | .84 | 55 | 56 | WSW | SW | | | fine |
| 17 | | | 48 | 56 | 45 | .83 | .80 | 56 | 53 | SSE | SSE | fog & sh. | | |
| 18 | | | 52 | 54 | 48 | .54 | .35 | 52 | 54 | SW | S | fine | | rain |
| 19 | | | 54 | 55 | 47 | .46 | .67 | 55 | 68 | W | W | | | cloudy |

The quantity of Rain fallen in the month of March, was 77.100ths of an inch.

